# Trends in metabolic signaling pathways of tumor drug resistance: A scientometric analysis

**DOI:** 10.3389/fonc.2022.981406

**Published:** 2022-10-25

**Authors:** Ruiqi Jiang, Mingnan Cao, Shenghui Mei, Shanshan Guo, Wei Zhang, Nan Ji, Zhigang Zhao

**Affiliations:** ^1^ Department of Pharmacy, Beijing Tiantan Hospital, Capital Medical University, Beijing, China; ^2^ Department of Clinical Pharmacology, College of Pharmaceutical Sciences, Capital Medical University, Beijing, China; ^3^ Department of Neurology, Beijing Tiantan Hospital, Capital Medical University, Beijing, China; ^4^ Department of Neurosurgery, Beijing Tiantan Hospital, Capital Medical University, Beijing, China

**Keywords:** scientometrics, tumor, metabolism, drug resistance, signaling pathways

## Abstract

**Background:**

Cancer chemotherapy resistance is one of the most critical obstacles in cancer therapy. Since Warburg O first observed alterations in cancer metabolism in the 1950s, people gradually found tumor metabolism pathways play a fundamental role in regulating the response to chemotherapeutic drugs, and the attempts of targeting tumor energetics have shown promising preclinical outcomes in recent years. This study aimed to summarize the knowledge structure and identify emerging trends and potential hotspots in metabolic signaling pathways of tumor drug resistance research.

**Methods:**

Publications related to metabolic signaling pathways of tumor drug resistance published from 1992 to 2022 were retrieved from the Web of Science Core Collection database. The document type was set to articles or reviews with language restriction to English. Two different scientometric software including Citespace and VOS viewer were used to conduct this scientometric analysis.

**Results:**

A total of 2,537 publications including 1,704 articles and 833 reviews were retrieved in the final analysis. The USA made the most contributions to this field. The leading institution was the University of Texas MD Anderson Cancer Center. Avan A was the most productive author, and Hanahan D was the key researcher with the most co-citations, but there is no leader in this field yet. *Cancers* was the most influential academic journal, and Oncology was the most popular research field. Based on keywords occurrence analysis, these selected keywords could be roughly divided into five main topics: cluster 1 (study of cancer cell apoptosis pathway); cluster 2 (study of resistance mechanisms of different cancer types); cluster 3 (study of cancer stem cells); cluster 4 (study of tumor oxidative stress and inflammation signaling pathways); and cluster 5 (study of autophagy). The keywords burst detection identified several keywords as new research hotspots, including “tumor microenvironment,” “invasion,” and “target”.

**Conclusion:**

Tumor metabolic reprogramming of drug resistance research is advancing rapidly. This study serves as a starting point, providing a thorough overview, the development landscape, and future opportunities in this field.

## Introduction

Drug resistance is prevalent in cancer treatment, and overcoming resistance is still one of the most pressing needs in cancer therapy (1). Drug resistance develops through a variety of mechanisms, including changes in drug transport and metabolism, mutation of the drug target, and activation of bypass survival pathways, which are frequently caused by tumor heterogeneity, allowing the escape and evolution of resistant cells (2). The intracellular physiology of drug-resistant cells is complex, with constant changes in energetic and oxidative-reductive metabolic signaling pathways (3).

The Warburg effect was first proposed by Warburg O in 1956 when he observed that many cancer cell lines relied on a high rate of glucose uptake and metabolism to maintain their viability despite being maintained in an oxygen-replete environment (4). It played an important role in tumor cell survival and proliferation, but the importance of metabolism in regulating drug resistance was only recently recognized (2). Scholars have now discovered some metabolic pathways of the Warburg effect and potential targets of these pathways that can improve drug resistance (5). In addition to glycolysis, other metabolic pathways of drug resistance have also been extensively studied in recent years, such as the pentose phosphate pathway (6), glutamine metabolism (7), and lipid metabolism (8).

In recent years, a significant number of researchers and academic journals have focused on reviewing relevant literature to summarize the current status of metabolic signaling pathways of tumor drug resistance. However, most of these papers have only centered on specific subfields of metabolic signaling pathways of tumor drug resistance (9, 10). For example, most reviews have paid attention to specific metabolic signaling pathways (11), autophagy (12), and targeted therapies (13). Little attention was paid to investigating the scientific output and current status systematically in this field from a global perspective, implying that there have been few reports focused on the scientometric perspective of this field. Therefore, it is critical to use an appropriate visualization method to reveal the global status, future research trends, and hotspots in this field.

Unlike systematic reviews, the scientometric analysis is a quantitative analysis of written scientific publications by analyzing data of these publications such as authors, countries, institutions, journals, and citations (14, 15). It may assist researchers in identifying core entities and development trends in a specific subject or research domain, as well as provide new insights and directions for future research (16).

At present, a variety of free tools have been developed for bibliometric mappings, such as Citespace and VOS viewer (15). CiteSpace V (Version 5.8 R3), a web-based Java application for data analysis and visualization, is an information visualization software tool created by Professor Chaomei Chen which visualizes the literature in the form of a co-citation network, which draws on article citations to reveal the structure of a field or fields (17). VOS viewer software is a scientometric visualization tool developed by Professor Eck and Waltman from Leiden University in the Netherlands using the Java language, which could visualize the knowledge structure, regularity, and distribution of scientific publications (18).

Rewiring of cell metabolism and bioenergetics is a characteristic feature of cancer development and has recently been recognized as a hallmark of cancer. However, the extent of the benefits these metabolic alterations provide to cancer cells is still not fully understood, particularly in terms of drug sensitivity and resistance. Even though current treatments can reduce tumor size and increase life expectancy, the main reason for the high mortality of cancer is the lack of effective treatments to overcome the natural acquisition of resistance (19). To the best of our knowledge, no previous study has specifically analyzed the knowledge structure and research frontiers in the field of metabolic signaling pathways of tumor drug resistance yet. As a result, this study is the first attempt to address this research gap. We use Citespace and VOS viewer to conduct a scientometric analysis of this field on the Web of Science Core Collection database from 1992 to 2022. This study aimed to (i) summarize the current research trends in this field, (ii) determine the knowledge structure, including the major academic groups and cooperation networks, (iii) identify the major participants and their collaboration networks, (iv) analyze research status and hotspots, (v) summarize the main research themes and clusters, and (vi) offer a new line of thinking.

## Materials and methods

### Data source

Data from the Science Citation Index Expanded (SCI-Expanded) database of the Web of Science Core Collection (WOS) was used in this study. The reasons for choosing this database are as follows. First of all, when compared to other databases such as PubMed and Embase, WOS is the most widely used, comprehensive, and authoritative tool for scientometric analysis across scientific disciplines (20). Following that, WOS is a typical citation database including citation and research collaboration information, facilitating scientometric analysis (21). Last but not least, the reference files provided by WOS could meet the specific format requirements as dictated by VOS viewer, Citespace, and other scientometric software (20, 22). An additional process is required to convert file format if downloaded from other databases. Given that our objective was to conduct a high-quality scientometric analysis to identify research trends in the core of metabolic signaling pathways of tumor drug resistance, WOS may be the only appropriate choice.

### Data collection

We performed online retrieval on 26 July 2022. Search terms used were chosen from the list of Medical Subject Headings (MeSH) and free text key terms were used as well. The following free terms were used: tumor, tumors, neoplasm, neoplasia, neoplasias, cancer, cancers, malignancy, malignancies, multidrug resistance, multiple drug resistance, metabolic pathways, metabolic pathway, metabolic networks, metabolic network, and metabolic signaling. The following MeSH terms were used: neoplasms, drug resistance, and metabolic networks and pathways. Therefore, the search terms and retrieval strategies were developed as follows: ((ALL= (neoplasias* OR tumor* OR cancer* OR malignanc*)) AND ALL= (drug resistance OR multidrug resistance OR multiple drug resistance)) AND ALL= (metabolic pathway* OR metabolic network* OR metabolic signaling). The MeSH term “Neoplasms” means new abnormal growth of tissue, malignant neoplasms show a greater degree of anaplasia and have the properties of invasion and metastasis, compared to benign neoplasms. Timespan: 1992 to 2022. The language type was limited to English. Original articles and reviews were included, while proceedings papers, early access, book chapters, editorial materials, meeting abstracts, and letters were excluded. The search retrieved a total of 2,537 records. Full records and cited references were downloaded in plain text format. Then the data files were imported into the software package CiteSpace (Version 5.8.R3) and VOS viewer (Version 1.6.18).

### Data analysis

CiteSpace and VOS viewer software were used for visual analysis, then Excel 2019 was used for quantitative analysis of the included literature. Co-authorship analysis refers to the evaluation of the relationship among items based on the number of coauthored documents, which were considered one of the most tangible indicators to evaluate collaboration trends and identify leading countries, institutions, and scientists (23, 24). Co-citation refers to when two or more authors or references are simultaneously cited in one or more subsequent papers, which is called a co-cited relationship between these two or more authors or references (25, 26). Author co-citation analysis can discover highly influential scholars in a discipline area. When references were frequently cited at the same time, indicating these studies were highly correlated and had similar research topics. The relatedness of co-occurrence analysis is determined based on the number of documents in which they occur together (24). The burst detection of references and keywords was based on Kleinberg’s algorithm, which can recognize sharp increases in scientific activity over a short time and capture the growing research interest in a specific research field (27).

Parameter Settings of CiteSpace: the period is from 1992 to 2022, the time zone is divided into one year, and the threshold (Top N) is set to 50, which means extracting the 50 nodes with the highest frequency every year. In the present study, this software was implemented for constructing network visualization of author co-authorship analysis, the co-occurring network of subject categories, as well as detecting the references and keywords with the strongest citation bursts.

Then, we use VOS viewer software to create co-authorship, co-citation, and co-occurrence networks in this field. Specifically, country co-authorship analysis, institution co-authorship analysis, author co-citation analysis, co-citation references analysis, and keyword co-occurrence analysis were performed and three visualization maps, including the network visualization map, the overlay visualization map, and the density visualization map were constructed in this study. Each node represents an individual country, and the node size is proportional to the number of publications. Line thickness between nodes indicates the link strength of a collaborative relationship and it is weighted by a quantitative evaluation indicator of total link strength (TLS). For a given item, the TLS attributes indicate the total strength of the links of an item with other items (28). For example, in the case of co-authorship links between researchers, the TLS attribute indicates the total strength of the co-authorship links of a given researcher with other researchers (29).

Apart from the above methods, an online visualization platform (https://www.datawrapper.de/) was also used to perform a geographical distribution map.

## Results and discussion

### Analysis of publications and citations

**Figure 1:**
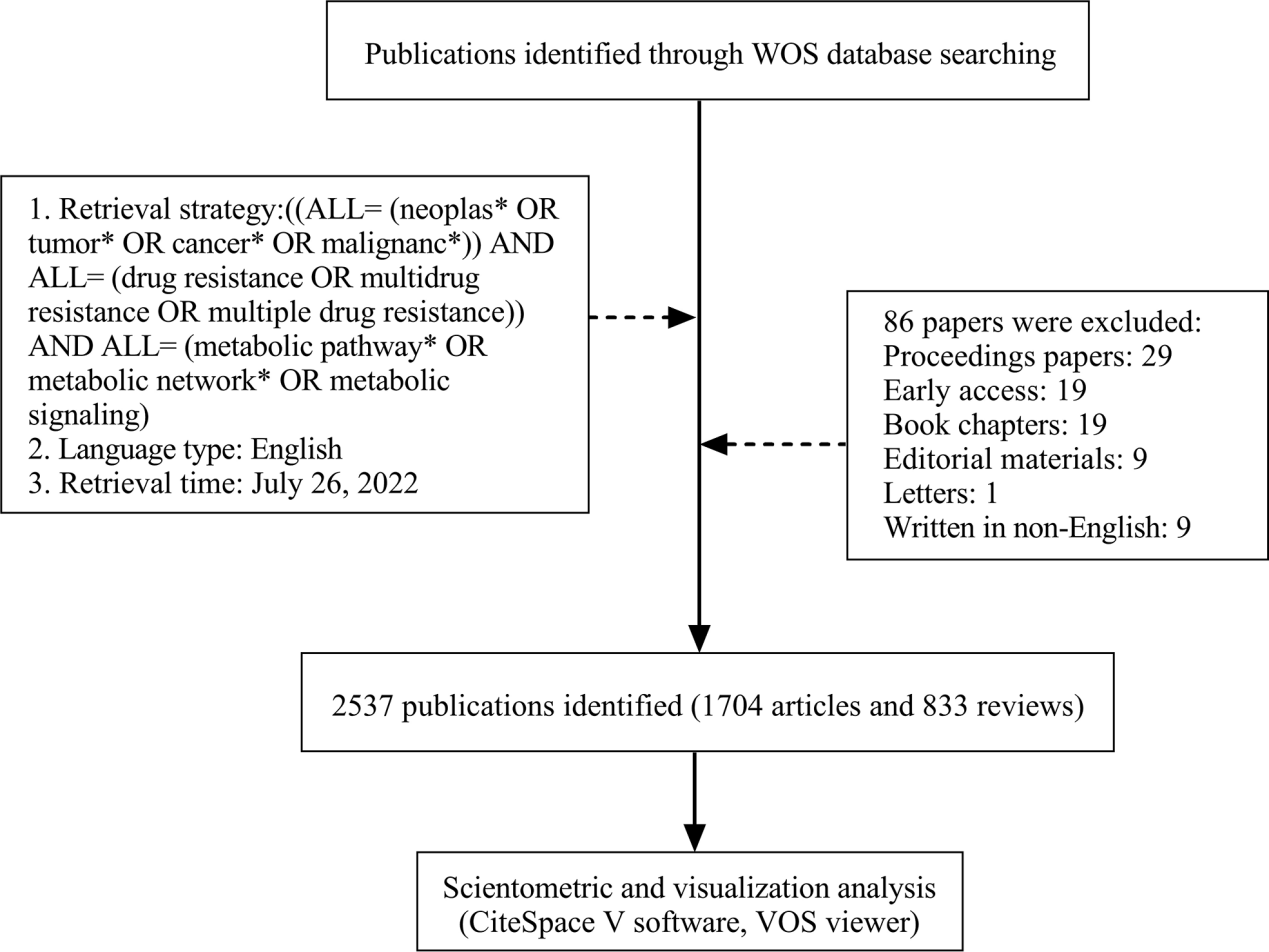
Flow chart of literature search and analysis.

**Figure 2:**
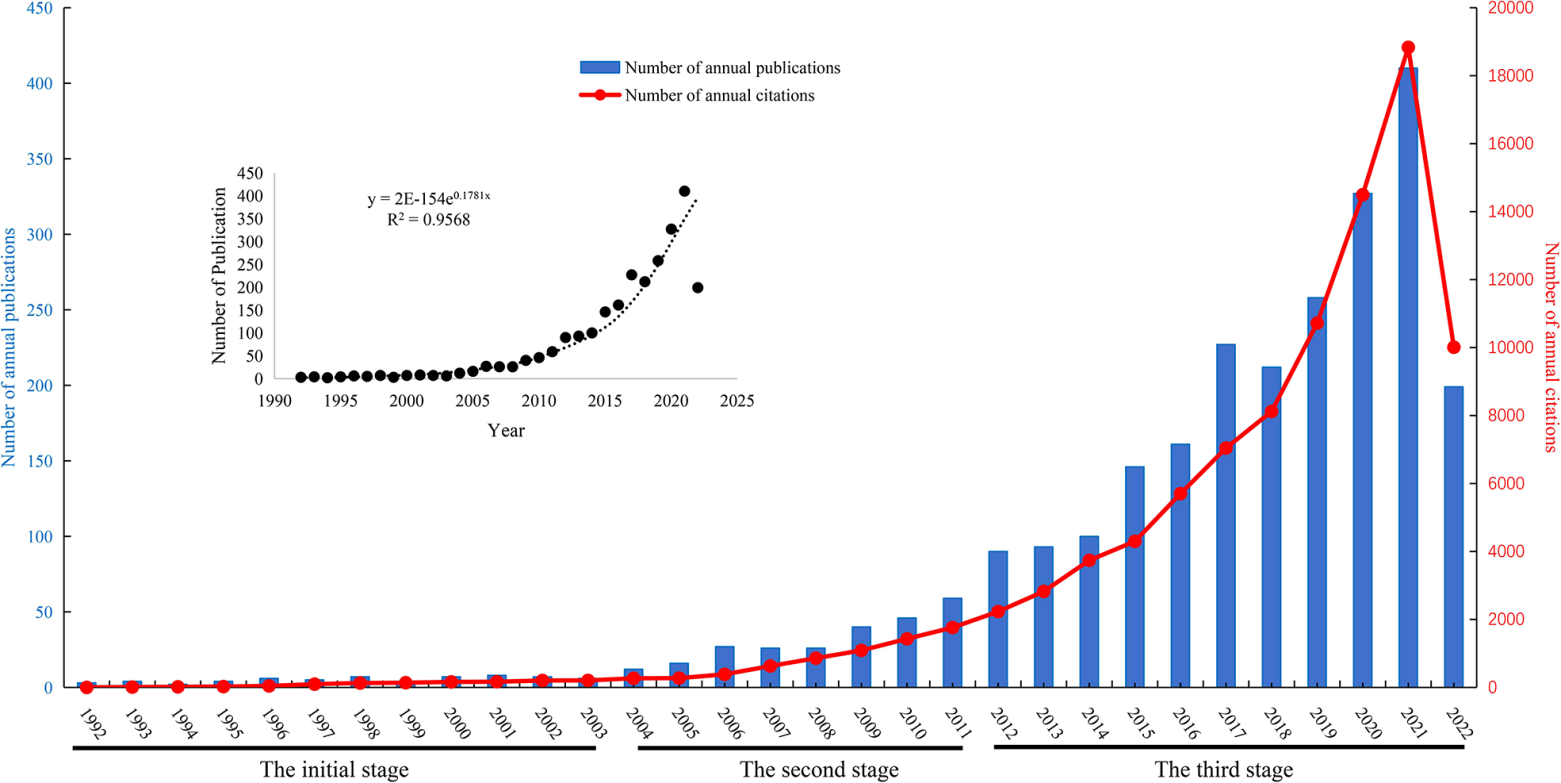
Distribution of the annual published documents and citations research related to metabolic signaling pathways of tumor drug resistance from 1992 to 2022.

A total of 2,537 publications in WOS including 1,704 articles and 833 reviews were collected from 1992 to 2022 for further analysis (**Figure 1**). The cumulative total citations for all articles were 96,002 times, including 2,102 times of self-citations by 26 July 2022, and an average of approximately 37.8 per document. A total of 8,053 publications in PubMed were collected from 1992 to 2022. The changing trend of annual publications and citations of published papers of WOS and annual publications of published papers of PubMed databases from 1992 to 2022 are shown in **Figures 2**, **S1**. Although PubMed databases could provide a broader range of coverage, much of the “extra coverage” could be attributed to journals with potentially limited readers (22). Research on metabolic signaling pathways of tumor drug resistance could be divided into three stages: the initial stage from 1992 to 2003, the second stage from 2004 to 2011, and the third stage from 2012 to 2022. The initial stage lasted for 12 years, during which the number of annual publications was less than 10. In the second stage, the number of annual publications increased slightly, but none exceeded 60. Since 2012, the number of annual publications had grown rapidly, and in 2014, the number of papers exceeded 100 for the first time. In addition, the total number of papers published in this period accounted for 87.62% of the papers included in the entire period. Moreover, the total citations showed a trend similar to that of annual publications. According to the current data, metabolic signaling pathways of tumor drug resistance is an emerging field with significant progress, and the interest of researchers in this field has surged. It can be predicted that more research on metabolic signaling pathways of tumor drug resistance will be published in the future.

### Country/region analysis

**Figure 3:**
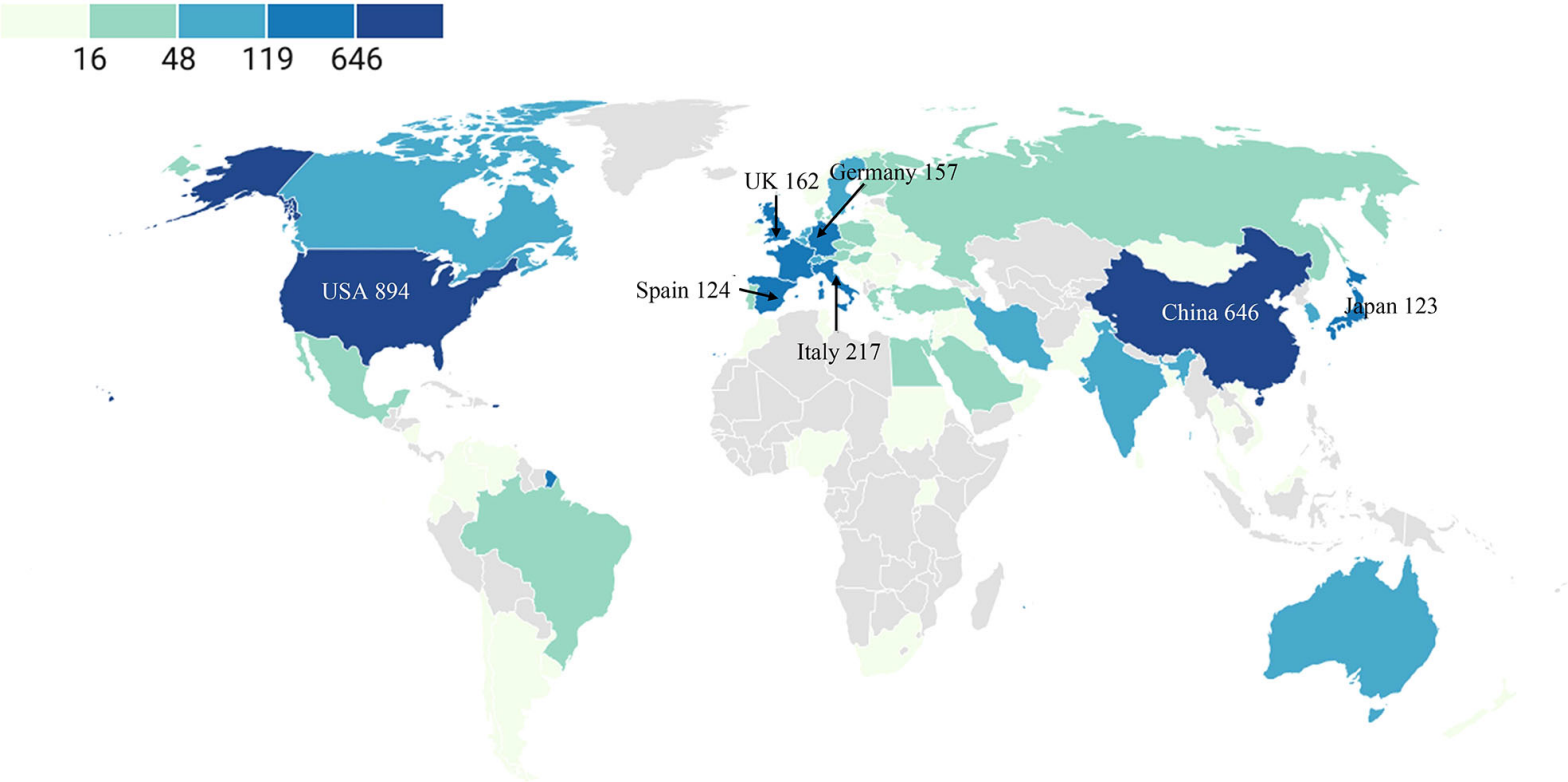
Geographic distribution map based on the total publications of different countries.

Of these 2,537 publications, 84 countries/regions have so far contributed to the publications on metabolic signaling pathways of tumor drug resistance research. Publications from Taiwan, Hong Kong, and Macau were assigned to China, and those from England, Northern Ireland, Scotland, and Wales were reclassified to the UK. As shown in **Table 1**, the top three countries with the most publications were the USA, China, Italy, and other countries that published <200 papers. A geographical distribution map based on the total publications of different countries is presented in **Figure 3**. In addition, considering that there were substantial differences in the number of populations among countries, which may have a direct impact on research achievements, another index of paper per million people was introduced for further analysis (24, 28). It can be used to compare author activity in different countries/regions. The latest demographic data (2021) were obtained from the World Bank official website (https://data.worldbank.org/indicator/SP.POP.TOTL) (30). After adjusting by population size, Sweden was on top with 4.80 papers per million people, followed by Italy (3.67) and the Netherlands (3.48), while the USA was only in the fifth position. Authors from North America, Eastern Asia, and Western Europe produced the majority of scientific publications in this field. The total amount of publications from these three regions were similar, and Western European authors were slightly more active than others.

**Table 1:** The top 15 productive countries contributed to publications on metabolic signaling pathways of tumor drug resistance research.

Ranking	Countries	Output [n (%)]	Population(in millions) (26)	Number of papers per million people	Optimized ranking	TLS	ACI
1	USA	894 (35.24%)	331.89	2.69	5	624	52.02
2	China	646 (25.46%)	1412.36	0.46	14	248	23.90
3	Italy	217 (8.55%)	59.07	3.67	2	194	37.65
4	UK	162 (6.39%)	67.33	2.41	7	248	47.09
5	Germany	157 (6.19%)	83.13	1.89	9	230	53.16
6	Spain	124 (4.89%)	47.33	2.62	6	129	40.63
7	Japan	123 (4.85%)	125.68	0.98	13	90	44.09
8	France	119 (4.69%)	67.50	1.76	10	154	33.93
9	India	94 (3.71%)	1393.41	0.07	15	62	21.21
10	Iran	85 (3.35%)	85.03	1.00	12	80	20.33
11	Canada	84 (3.31%)	38.25	2.20	8	97	40.95
12	Australia	79 (3.11%)	25.74	3.07	4	101	52.03
13	South Korea	73 (2.88%)	51.74	1.41	11	41	27.63
14	Netherlands	61 (2.40%)	17.53	3.48	3	85	42.44
15	Sweden	50 (1.97%)	10.42	4.80	1	102	42.10

TLS, Total link strength; ACI, Average citations per item.

In addition, **Figure S2** illustrates the co-authorship among countries’ visualization map. As we can see from the map, active collaboration was observed between prolific countries. For example, the USA collaborated closely with China and Spain. The top three with the largest TLS countries were the USA, the UK, and China.

### Journal analysis

We have listed the top 10 most productive journals on metabolic signaling pathways of tumor drug resistance research in **Table S1**. These 10 prolific journals published 486 papers, constituting 19.16% of all 2,537 publications. Among the top 10 journals, the most noteworthy journal was *Cancers*, followed by *Frontiers in Oncology*, and *International Journal of Molecular Sciences*. It is not difficult to find that research outputs are mainly published in these journals focused on Oncology and Pharmacy. According to the 2021 Journal Citation Reports (JCR) reported, the impact factor (IF) of all the top 10 journals ranged from 3.752 (*Plos One*) to 13.312 (*Cancer Research*), and all journals were categorized in Q1 or Q2 except *Oncotarget*. Moreover, all the top 10 journals were hosted by North American (the USA) and Western European (England and Switzerland) countries, which might be one of the important factors for Euro-American countries to dominate this field.

### Author analysis

**Table S2** shows the top 20 authors according to the publication numbers in this field. Most of these authors came from the USA, Iran, and China. Avan A from Mashhad University Medical Science was the most productive author with 19 papers, followed by Mirzaei H from Kashan University of Medical Sciences with 14 papers. Most authors only contributed one or two papers, indicating that a few authors made sustained contributions to this field. The authors’ co-authorship relationship network map is demonstrated in **Figure S3A**. The centrality index for each author was 0.0, and a quite small number of connection links were shown in the network map, which reflected that the collaboration between different research teams was not very common. Therefore, it’s necessary to improve international communication between research groups to promote the development of this field.

What’s more, collaborative maps of co-cited authors contributed to publications on metabolic signaling pathways of tumor drug resistance research by VOS viewer are shown in **Figure S3B**, and the top 20 co-cited authors are listed in **Table S3**. The color of the nodes and lines indicated different appearance clusters. The node size is proportional to citation frequency. A line between two nodes indicates that both were cited by one author. The association between items is created based on the number of times they are cited jointly by a third citing item, which is known as co-citation analysis (31). As can be seen from **Figure S3B**, co-cited authors could be roughly divided into four clusters. The authors in Cluster 1 (red nodes) focused on anticancer therapy targeting cancer cell metabolism, especially colorectal cancer (32); the authors in cluster 2 (green nodes) focused on the Warburg effect (33); the authors in cluster 3 (blue nodes) focused on autophagy dysfunction in cancer (34); the authors in cluster 4 (yellow nodes) focused on aging and cancer (35). The top 3 authors with the greatest TLS were as follows: Hanahan D, Warburg O, and Kim J. Compared with the top 20 productive authors, no author was included in the list of the top 20 co-cited authors, indicating that there is no leading figure in this field yet.

**Table S4** illustrates the top 20 authors with the strongest citation bursts from 1992 to 2022. The top-ranked author by bursts was Jemal A and followed by Gatenby RA and Laplante M. Gottesman MM (36) was the first author who received special attention in this field in 2003. Bray F worked on cancer epidemiology (37), which was the most recent burst and has lasted for 2 years now.

### Institution analysis

As for institutions analysis, the top 15 most prolific institutions and the number of publications in each institution are presented in **Figure S4**. The top 15 institutions, with 476 published articles, accounted for 20.13% of total publications. Among these six institutions originated from North America, seven from Asia, and two came from Western Europe. The most prolific individual institution in terms of the number of publications was the University of Texas MD Anderson Cancer Center, followed by the National Cancer Institute and the Chinese Academy of Sciences. This may be one of the reasons for the high number of papers published in these regions.

Besides, the institution citation analysis was performed by VOS viewer (**Figure S5**). The top 3 institutions with the largest TLS were Sun Yat-sen University, the University of Texas MD Anderson Cancer Center, and the University of Florence. Surprisingly, Sun Yat-sen University and the University of Florence were not the top 3 productive institutions, but they still had such high impacts, indicating that they had performed a lot of high-quality research. In addition, the University of Texas MD Anderson Cancer Center was the most productive and influential institution.

### Subject category analysis

**Figure 4:**
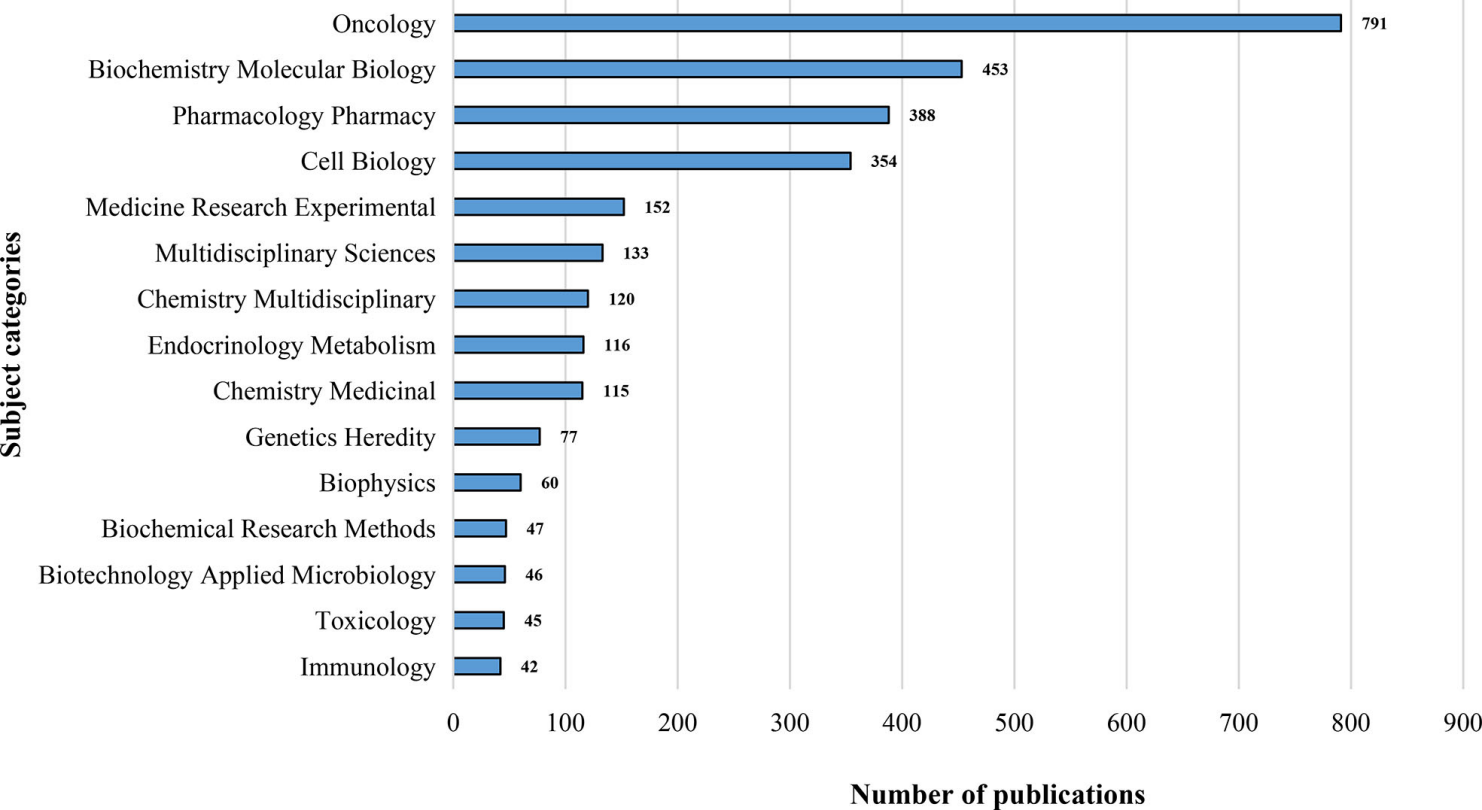
The top 15 subject categories related to metabolic signaling pathways of tumor drug resistance.

Depending on the content classification of the Web of Science database, the study of metabolic signaling pathways of tumor drug resistance was distributed across 89 specific subject categories. The top 15 research areas covered by the leading journals are illustrated in **Figure 4**. The most represented categories based on the number of publications were Oncology, Biochemistry and Molecular Biology, and Pharmacology/Pharmacy. The co-occurring subject categories network of this field by using CiteSpace is mapped in **Figure S6**. A purple ring on the edge of the node represents that this node has a high betweenness centrality, in other words, indicates its critical role in bridging the nodes it links. The top five subject categories ranked by centrality were Biochemistry and Molecular Biology (0.46), Oncology (0.40), Pharmacology/Pharmacy (0.30), Cell Biology (0.19), and Biotechnology and Applied Microbiology (0.12). Taking the above results into consideration, it can be noted that intranasal delivery research involves multiple disciplines. Pharmacology/Pharmacy and Neurosciences/Neurology were the hottest research categories in this field. Research on metabolic signaling pathways of tumor drug resistance has covered multidisciplinary knowledge and interdisciplinary collaboration among different fields might help to improve scientific work and maximize the potential.

### Research topics and clusters of global publications

#### Analysis of highly co-cited references

High-cited publications usually refer to high-quality studies with significant influence and innovation in a certain subject and have received greater attention in this field (38). The details of the top 10 co-citations of original articles related to metabolic signaling pathways of tumor drug resistance are shown in **Table 2**. Three of the top 10 references were published in 2013, and others were published before 2010. All of them were co-cited less than 50 times. Among them, the most highly cited reference was an article by Subramanian A (39) with 49 times. They described a powerful analytical method called Gene Set Enrichment Analysis (GSEA) for interpreting gene expression data. The common tumor signatures are closely associated with various tumor types. Tumor signatures identified through the gene set enrichment analysis based on gene expression profiling could help to establish and validate metabolism-related prognostic models, discovering drug resistance signaling pathways (40). Notably, the eighth article (41) provided another gene lists analysis protocol explaining how to use DAVID bioinformatics resources, which consisted of an integrated biological knowledge base and analytic tools aimed at systematically extracting biological meaning from large gene/protein lists.

**Table 2:** The top 10 co-citation of original articles.

Ranking	Title	Total citations	Journal	First author	Year
1	Gene set enrichment analysis: a knowledge-based approach for interpreting genome-wide expression profiles	49	Proceedings of the National Academy of Sciences of The United States of America	Subramanian A	2005
2	HIF-1-mediated expression of pyruvate dehydrogenase kinase: a metabolic switch required for cellular adaptation to hypoxia	47	Cell Metabolism	Kim JW	2006
3	Oncogenic BRAF regulates oxidative metabolism via PGC1α and MITF	46	Cancer Cell	Haq R	2013
4	Glutamine supports pancreatic cancer growth through a KRAS-regulated metabolic pathway	41	Nature	Son J	2013
5	PGC1α expression defines a subset of human melanoma tumors with increased mitochondrial capacity and resistance to oxidative stress	41	Cancer Cell	Vazquez F	2013
6	The M2 splice isoform of pyruvate kinase is important for cancer metabolism and tumour growth	40	Nature	Christofk HR	2008
7	c-Myc suppression of miR-23a/b enhances mitochondrial glutaminase expression and glutamine metabolism	40	Nature	Gao P	2009
8	Systematic and integrative analysis of large gene lists using DAVID bioinformatics resources	40	Nature Protocols	Huang da W	2009
9	Myc regulates a transcriptional program that stimulates mitochondrial glutaminolysis and leads to glutamine addiction	40	Proceedings of the National Academy of Sciences of The United States of America	Wise DR	2008
10	Inhibition of glycolysis in cancer cells: a novel strategy to overcome drug resistance associated with mitochondrial respiratory defect and hypoxia	40	Cancer Research	Xu RH	2005

Other papers (42–49) all focused on the Warburg effect, which referred to many cancer cell lines depending on a high rate of glucose uptake and metabolism to maintain their viability despite being maintained in an oxygen-replete environment (33). This metabolic phenotype had been termed aerobic glycolysis. Xu RH (49) proposed that depletion of ATP by glycolytic inhibition induced apoptosis in multidrug-resistant cells, therefore, deprivation of cellular energy supply might be an effective way to overcome multidrug resistance. Vazquez F (47) and Haq R (44) reported that the oncogenic melanocyte lineage-specification transcription factor MITF drove PGC1α overexpression in a subset of human melanomas and derived cell lines. In addition, Haq R (44) found that melanomas with activation of the BRAF/MAPK pathway had suppressed levels of MITF and PGC1α, and decreased oxidative metabolism. Gao P (43) and Wise DR (48) described that oncogenic levels of Myc induced a transcriptional program that promoted glutaminolysis and triggered cellular addiction to glutamine as a bioenergetic substrate.

Moreover, a co-citation network visualization map of references generated by the VOS viewer was shown in **Figure S7**. The color of the nodes and lines indicated different appearance clusters. The node size is proportional to citation frequency. A line between two nodes indicates that both were cited by one reference. The co-citation references could be roughly divided into five main topics: cluster 1 (red nodes) study of signaling pathways in the mutated gene in human cancer (41); cluster 2 (green nodes) study of the Warburg effect (50); cluster 3 (blue nodes) study of oxidative stress (51); cluster 4 (yellow nodes) study of cell metabolism regulator (52); cluster 5 (violet nodes) study of glutamine metabolism to cancer therapy (48). The reference of Hanahan D was located in central positions within the network. The review by Hanahan D published in 2011 (53) revisited, refined, and extended the concept of cancer hallmarks, and provided a useful conceptual framework for understanding the complex biology of cancer. They proposed that reprogramming energy metabolism was an emerging hallmark, and the role of aerobic glycolysis in malignant growth would be elucidated during the coming decade. The review by Vander Heiden MG published in 2009 (50) was another highly co-cited reference. They proposed that the metabolism of cancer cells, and indeed all proliferating cells, was adapted to facilitate the uptake and incorporation of nutrients into the biomass needed to produce a new cell.

#### Analysis of references with citation burst

References with citation bursts can be used to examine thematic trends due to bias between old and newly published literature. A citation burst shows that academia has paid extra attention to cited papers (54). **Table S5** illustrates the top 25 references (44, 50, 53, 55–76) with the strongest citation bursts from 1992 to 2022 conducted through CiteSpace. The minimum duration of the burst was set for five years, and a red line segment represented the initial and final years of the burst duration. Reference with citation burst was first observed in 2011, which is due to a review by Vander Heiden MG in 2009 (50). This review related to Warburg Effect was also the second most highly cited reference which indicated that this article had received special attention from associated academic circles in the past period. The most recent burst appeared in 2020 and has lasted for 1 year by now. The publication reviewed known cancer-associated metabolic changes into six hallmarks that have been identified as the strongest burst references since 2017 and the burst remains ongoing (55). Glycolysis and lipid metabolism were two metabolic pathways that attracted more attention, and researchers had discovered some drug resistance targets in these metabolic pathways, such as MYC and MCL1 cooperated in the maintenance of triple-negative breast cancer (56) and JAK/STAT3 regulated lipid metabolism and promoted breast cancer chemoresistance (57). In addition, cancer statistics were another topic of high interest (58–60). This reflects the importance and necessity of studying metabolic signaling pathways of tumor drug resistance and may be related to the major scientific progress made in this field in recent years.

#### Analysis of co-occurrence keywords

Analyzing the results of keywords co-occurrence can identify the research hotspots and frontiers of metabolic signaling pathways of tumor drug resistance. The keyword co-occurrence visualization map was constructed with VOS viewer. A density visualization map is shown in **Figure S8**, “drug resistance” was the most frequent keyword, followed by “cancer”, “expression”, “resistance”, and “metabolism”. This was consistent with our research theme. To further investigate the topic structure of metabolic signaling pathways of tumor drug resistance research, we also used CiteSpace to draw a keyword co-occurrence visualization map (**Figure S9**).

**Figure 5:**
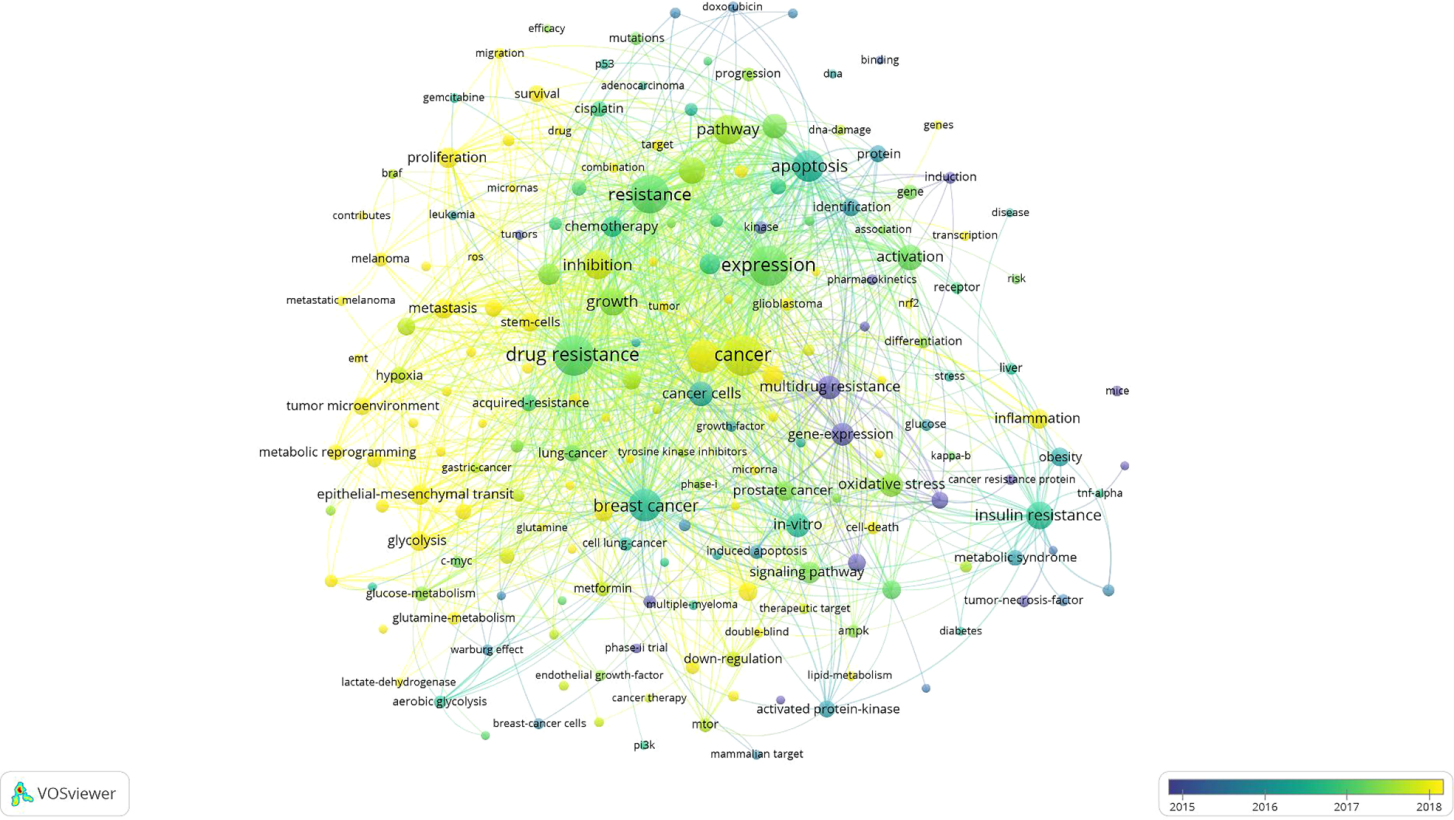
Overlay visualization map of keyword co-occurrence analysis. The node and label meanings in this map are the same as in the network visualization map. Furthermore, the color of a node indicates the APY of each keyword according to the color gradient shown in the lower right corner.

In addition, the overlay visualization map and network visualization map of keyword co-occurrence analysis are shown in **Figures 5**, **S10**, respectively. As can be seen from **Figure S10**, 192 keywords that occurred at least 20 times could be roughly divided into five main topics: cluster 1 (red nodes, upper right of the network); cluster 2 (green nodes, bottom side of the network); cluster 3 (blue nodes, bottom left of the network); cluster 4 (yellow nodes, bottom right of the network); cluster 5 (violet nodes, upper left of the network).

##### Cluster 1: Study of cancer cell apoptosis pathway

Cluster 1 was the largest cluster with 48 keywords, and the primary keywords were cancer, expression, resistance, apoptosis, pathway, inhibition, growth, cells, activation, and mechanism. Several researchers have investigated that intracellular apoptosis pathways in human cancers abnormally activated significantly promoted tumor progression, regulation of cell metabolism, and the development of drug resistance to chemotherapeutics (77). For example, Nuan-Aliman, S (78). reported that the alternative NF-κB pathway impacted diffuse large B-cell lymphoma cell energy homeostasis enhanced oxidative phosphorylation energy metabolism, and drove resistance of cells to apoptosis induced by mitochondrial metabolic stress upon treatment with two approved anti-metabolic drugs, or glutamine deprivation. Therefore, finding inhibitors that exerted a targeted therapeutic effect is an efficient method to relieve apoptosis inhibition and drug resistance, such as HSF1 inhibitor KRIBB11 (79), BCL-2 inhibitor Venetoclax (80), and ligustrazine-curcumin hybrids 10d (81).

##### Cluster 2: Study of resistance mechanisms of different cancer types

Cluster 2 was the second-largest cluster with 46 keywords. This cluster mainly focused on resistance mechanisms of different cancer types including the following keywords: *in-vitro*, multidrug resistance, hepatocellular carcinoma, colorectal cancer, tumor microenvironment, p-glycoprotein, acquired resistance, down-regulation, acute myeloid leukemia, and cell lung cancer. The resistance mechanisms of cancer include changes in drug transport and metabolism and mutation of the drug target (61). Among mechanisms of resistance, multidrug resistance is a process independent of the chemical structures of drugs that reduce intracellular drug accumulation. It’s often due to upregulation and/or amplification of the MDR1/ABCB1 gene that encodes p-glycoprotein (82). Some researchers developed a particular class of hybrid by conjugating anticancer agents with nitric oxide (NO) donors, the appropriate NO donors reversed drug resistance *via* nitration of ABC transporters, and by interfering with several metabolic enzymes and signaling pathways (83). Hepatocellular carcinoma is an aggressive human cancer with increasing incidence worldwide and its resistance becomes a research hotspot. Ren Y (84) proposed that CXCR3 induced metabolic alteration in SOR-resistance hepatocellular carcinoma cells through downregulating AMPK pathway activity and lipid peroxidation as well as upregulating levels of adipocytokines. In addition, some components (such as YAP/TAZ molecules and hypoxia-inducible factor-1) in the tumor microenvironment involved in mediating the altered metabolic pathways appear to be critical therapeutic targets that can be used to enhance current cancer treatment for metastatic and treatment-resistant cancers (85, 86).

##### Cluster 3: Study of cancer stem cells

Cluster 3 contained 45 keywords, mainly involved in metabolic of cancer drug resistance studies: drug resistance, breast cancer, epithelial-mesenchymal transition, metastasis, glycolysis, prostate cancer, stem cells, mitochondria, hypoxia, lung-cancer, chemoresistance, cancer metabolism, and metabolic reprogramming. In the presence of oxygen, cancer stem cells can switch between oxidative phosphorylation and glycolysis to maintain homeostasis (87). Due to their highly tumorigenic and drug-resistant properties, dual inhibition of metabolic pathways may be an effective therapeutic strategy for eradicating heterogeneous cancer stem cells rather than singularly targeting glycolysis or oxidative phosphorylation pathways (87, 88). In addition, cancer metastatic ability was closely associated with cancer stem cell phenotype, especially the process known as epithelial-mesenchymal transition, therefore, epithelial-mesenchymal transition plays a critical role in driving tumor metastatic dissemination and drug resistance (89). For example, a study (90) reported that podocalyxin-like protein 1 (PODXL) was induced during the epithelial-mesenchymal transition process, and it was a metabolic reprogramming inducer that enhanced glutamine metabolism and lipid metabolism, thereby increasing the proliferation of Raji Burkitt lymphoma cells. What’s more, overexpression of PODXL in Raji Burkitt lymphoma cells decreases dexamethasone- and hydrogen peroxide-induced cell apoptosis (91). With a further understanding of cancer stem cells’ metabolic pathways and principal players of metabolism, more potential therapeutic targets will be explored to improve cancer treatments.

##### Cluster 4: Study of tumor oxidative stress and inflammation signaling pathways

Cluster 4 contained 39 keywords, mainly focused on tumor oxidative stress and inflammation signaling pathways: metabolism, insulin resistance, oxidative stress, gene expression, signaling pathway, inflammation, NF-κB, obesity, *in-vivo*, and activated protein-kinase. Oxidative stress and inflammation damage lead to cancer initiation and progression (92). Overproduction of reactive oxygen species is linked to metabolic imbalance, and it has the potential to activate pro-oncogenic signaling pathways, change gene expression, and facilitate mutations, DNA damage, and genomic instability (93). In addition, reactive oxygen species and other overabundant radicals also lead to the activation of certain transcription factors and the hyperproduction of informative pro-inflammatory molecules (94). These processes can all switch healthy cells’ regular metabolism to the creation of a tumor-like state. NF-κB is currently a research hotspot. NF-κB participates in the modulation of the inflammatory response and can be activated by the effects of oxidative stress (95). Its activation is closely related to the development and progression of malignant neoplasms, for example, NF-κB can change the metabolism of tumor cells from mitochondrial-dependent oxidative phosphorylation to anaerobic glycolysis, which leads to the emergence of the Warburg effect and the adaptation of cancer cells to hypoxia conditions (96). Sanchez-Lopez E (97) found that the activation of NF-κB dependent p62-NRF2 cascade contributed to the survival and therapy resistance of chronic lymphocytic leukemia cells that expressed high levels of ROR1. What’s more, Zhang J (98) reported that TPH-1 facilitated cellular proliferation, migration, and chemoresistance in glioma through the serotonin/L1CAM/NF-κB pathway. Therefore, these targets may be effective strategies for the treatment of drug resistance.

##### Cluster 5: Study of autophagy

Cluster 5 was the smallest cluster with 14 keywords regarding autophagy. The main keywords in this cluster were: autophagy, proliferation, pancreatic cancer, progression, invasion, migration, overexpression, and gemcitabine. Autophagy is a double-edged sword: it suppresses tumorigenesis in the early stage and turns into an accomplice by controlling energy metabolism while cancer is established (99). Autophagy and mitochondrial metabolism collaborate in cancer cells to sustain their survival and proliferation in favor of tumor growth (100). Therefore, autophagy is of particular importance for cancers, such as pancreatic ductal adenocarcinoma. Reyes-Castellanos G (101) proposed that high autophagic activity in pancreatic ductal adenocarcinoma is markedly related to resistance to current therapies. The inhibitions in the last stage of autophagy can overcome drug resistance, such as chloroquine and its derivative hydroxychloroquine (102).

##### The transition of research focus

As we can see from **Figure 5**, the nodes coded with dark violet represent the keywords that appeared relatively earlier in the time course before or around 2015, while keywords that appeared around 2018 were coded with yellow color. It can be seen by combining the specific position of five clusters from **Figure 5** that early research around the year 2011, “study of tumor oxidative stress and inflammation signaling pathways and resistance mechanisms of different cancer types (cluster 4 and cluster 2)” had attracted a lot of attention in academia of this field. Afterward, “Study of cancer cell apoptosis pathway (cluster 1)” progressively gained importance around 2014, and some areas remain the hotpots until today, for example, “androgen receptor” (APY = 2018.82) (103), “glioblastoma” (APY= 2018.54) (79), and “transcription” (APY= 2018.15) (104). Notably, cluster 3 and cluster 5 (“study of cancer stem cells and autophagy”) are the two clusters that had the smallest APY compared with other clusters, and “metabolic reprogramming” (APY= 2019.39) (105), “microRNAs” (APY= 2019.05) (106) were mainly found lately. This implies that these two clusters are emerging topics in recent years, and more related research may be published in the future. These results suggest that the research focus of this field has switched from “cluster 4 and cluster 2: the study of tumor oxidative stress and inflammation signaling pathways and resistance mechanisms of different cancer types” to “cluster 3 and cluster 5: the study of cancer stem cells and autophagy”.

#### New hotspots and research frontiers

Burst detection was used to identify keywords that drew the attention of peer scholars over a specific period. It is also usually regarded as an important metric for determining research hotspots, developing trends, and research frontiers over time (107). We have analyzed the significant burst keywords between 1992 through 2022 by using CiteSpace, the top 25 keywords with the strongest citation bursts were shown in **Table 3**. As we can see from the overall change tendency of burst keywords, the research hotspot has experienced a transition to precision cancer medicine. Besides, it’s interesting to note that three burst keywords including “tumor microenvironment” (2020–2022) (85), “invasion” (2019-2022) (108), and “target” (2019–2022) (109) are still ongoing. Metabolic signatures within the tumor microenvironment impact the immune function, therefore, overcoming individualized microenvironment-related resistance and identifying related novel targets and signatures will be research hotspots in the future (110). As a result, we can expect more research into these areas in the future, leading to even more exciting scientific discoveries.

**Table 3:** Top 25 keywords with the strongest citation bursts.

Keywords	Strength	Begin	End	1992-2021
gene expression	10.95	2007	2013	▂▂▂▂▂▂▂▂▂▂▂▂▂▂▂▃▃▃▃▃▃▃▂▂▂▂▂▂▂▂▂
tumor microenvironment	10.4	2020	2022	▂▂▂▂▂▂▂▂▂▂▂▂▂▂▂▂▂▂▂▂▂▂▂▂▂▂▂▂▃▃▃
in vivo	9.25	2004	2017	▂▂▂▂▂▂▂▂▂▂▂▂▃▃▃▃▃▃▃▃▃▃▃▃▃▃▂▂▂▂▂
tumor necrosis factor	9.1	1998	2013	▂▂▂▂▂▂▃▃▃▃▃▃▃▃▃▃▃▃▃▃▃▃▂▂▂▂▂▂▂▂▂
cancer resistance protein	7.89	2009	2016	▂▂▂▂▂▂▂▂▂▂▂▂▂▂▂▂▂▃▃▃▃▃▃▃▃▂▂▂▂▂▂
molecular mechanism	7.32	2018	2020	▂▂▂▂▂▂▂▂▂▂▂▂▂▂▂▂▂▂▂▂▂▂▂▂▂▂▃▃▃▂▂
multidrug resistance	6.84	2003	2013	▂▂▂▂▂▂▂▂▂▂▂▃▃▃▃▃▃▃▃▃▃▃▂▂▂▂▂▂▂▂▂
induction	5.81	2004	2010	▂▂▂▂▂▂▂▂▂▂▂▂▃▃▃▃▃▃▃▂▂▂▂▂▂▂▂▂▂▂▂
p glycoprotein	5.73	2006	2013	▂▂▂▂▂▂▂▂▂▂▂▂▂▂▃▃▃▃▃▃▃▃▂▂▂▂▂▂▂▂▂
activated protein kinase	5.7	2010	2014	▂▂▂▂▂▂▂▂▂▂▂▂▂▂▂▂▂▂▃▃▃▃▃▂▂▂▂▂▂▂▂
transcription factor	5.13	2014	2016	▂▂▂▂▂▂▂▂▂▂▂▂▂▂▂▂▂▂▂▂▂▂▃▃▃▂▂▂▂▂▂
induced apoptosis	4.78	2012	2016	▂▂▂▂▂▂▂▂▂▂▂▂▂▂▂▂▂▂▂▂▃▃▃▃▃▂▂▂▂▂▂
obesity	4.59	2016	2017	▂▂▂▂▂▂▂▂▂▂▂▂▂▂▂▂▂▂▂▂▂▂▂▂▃▃▂▂▂▂▂
pharmacokinetics	4.56	2011	2016	▂▂▂▂▂▂▂▂▂▂▂▂▂▂▂▂▂▂▂▃▃▃▃▃▃▂▂▂▂▂▂
invasion	4.54	2019	2022	▂▂▂▂▂▂▂▂▂▂▂▂▂▂▂▂▂▂▂▂▂▂▂▂▂▂▂▃▃▃▃
growth factor receptor	4.45	2011	2015	▂▂▂▂▂▂▂▂▂▂▂▂▂▂▂▂▂▂▂▃▃▃▃▃▂▂▂▂▂▂▂
phosphorylation	4.43	2014	2016	▂▂▂▂▂▂▂▂▂▂▂▂▂▂▂▂▂▂▂▂▂▂▃▃▃▂▂▂▂▂▂
poor prognosis	4.39	2018	2020	▂▂▂▂▂▂▂▂▂▂▂▂▂▂▂▂▂▂▂▂▂▂▂▂▂▂▃▃▃▂▂
adipose tissue	4.38	2008	2012	▂▂▂▂▂▂▂▂▂▂▂▂▂▂▂▂▃▃▃▃▃▂▂▂▂▂▂▂▂▂▂
metabolic syndrome	4.24	2008	2012	▂▂▂▂▂▂▂▂▂▂▂▂▂▂▂▂▃▃▃▃▃▂▂▂▂▂▂▂▂▂▂
skeletal muscle	4.16	2008	2014	▂▂▂▂▂▂▂▂▂▂▂▂▂▂▂▂▃▃▃▃▃▃▃▂▂▂▂▂▂▂▂
breast	4.13	2012	2016	▂▂▂▂▂▂▂▂▂▂▂▂▂▂▂▂▂▂▂▂▃▃▃▃▃▂▂▂▂▂▂
differentiation	4.07	2015	2017	▂▂▂▂▂▂▂▂▂▂▂▂▂▂▂▂▂▂▂▂▂▂▂▃▃▃▂▂▂▂▂
messenger rna	4.01	2010	2015	▂▂▂▂▂▂▂▂▂▂▂▂▂▂▂▂▂▂▃▃▃▃▃▃▂▂▂▂▂▂▂
target	3.96	2019	2022	▂▂▂▂▂▂▂▂▂▂▂▂▂▂▂▂▂▂▂▂▂▂▂▂▂▂▂▃▃▃▃

## Limitations

There are some limitations to this study. Firstly, our study is only based on the WOS database, it is possible to overlook some relevant publications from other databases. However, it can’t combine data from different databases because the methods of calculating citations of different databases are various. PubMed does not provide references for an article as part of its metadata, the references cited by an article are not readily available from the dataset, which prevents researchers from conducting citation-based network analyses (29, 111). For example, data downloaded from PubMed cannot be used for identifying citation, bibliographic coupling, and co-citation links between items by VOS viewer and CiteSpace. When the literature is included and excluded, because the article type classification of PubMed is more complicated, it is difficult to directly include research articles and reviews like WOS. Therefore, the literature included in PubMed will have some deviations. Besides, most previous scientometric studies used only one database (21, 107). Secondly, our retrieval time is up to July 26th, since there are constantly new papers published, our research cannot include the recently published articles. Thirdly, our study only included English publications, which may be slightly biased for the research results, but non-English papers could not be included in scientometric studies due to the lack of references and other reasons (21).

## Conclusion

For the first time, the total knowledge framework and current state of metabolic signaling pathways of tumor drug resistance research were visualized using a scientometric method in this study. The rapid development of this field was confirmed in publications between 1992 and 2022. Since 2012, the number of annual publications had grown rapidly, and in 2014, the number of papers exceeded 100 for the first time, indicating a surge of interest in this field over the previous decades. The USA made the most contributions to this field. The leading institution was the University of Texas MD Anderson Cancer Center. Avan A was the most productive author, and Hanahan D was the key researcher with the most co-citations, but there is no leader in this field yet. *Cancers* was the most influential academic journal, and Oncology was the most popular research field. The co-citation references could be roughly divided into five main topics: cluster 1 (signaling pathways in the mutated gene in human cancer); cluster 2 (the Warburg effect); cluster 3 (oxidative stress); cluster 4 (cell metabolism regulator); cluster 5 (glutamine metabolism to cancer therapy). Based on keywords occurrence analysis, these selected keywords could be roughly divided into five main topics: cluster 1 (study of cancer cell apoptosis pathway); cluster 2 (study of resistance mechanisms of different cancer types); cluster 3 (study of cancer stem cells); cluster 4 (study of tumor oxidative stress and inflammation signaling pathways); and cluster 5 (study of autophagy). Concerning the APY of the keywords, it can be concluded that precision cancer medicine may be the future frontier of this field. The keywords burst detection identified several keywords as new research hotspots, including “tumor microenvironment,” “invasion,” and “target”. In a word, this study provides a comprehensive scientometric analysis of metabolic signaling pathways of tumor drug resistance research from a global perspective, and it can serve as a starting point for researchers and policymakers worldwide to identify and contribute to the growing scientific work in this field.

## Data availability statement

The original contributions presented in the study are included in the article/**Supplementary Material**. Further inquiries can be directed to the corresponding authors.

## Author contributions

RJ, MC, SM, SG, WZ, NJ, and ZZ designed the study. RJ collected the data. RJ and MC analyzed the data and drafted the manuscript. RJ, MC, SM, SG, WZ, NJ, and ZZ revised and approved the final version of the manuscript. All authors contributed to the article and approved the submitted version.

## Funding

This study was supported by Capital Health Research and Development of Special Grant (2022-2-2047).

## Conflict of interest

The authors declare that the research was conducted in the absence of any commercial or financial relationships that could be construed as a potential conflict of interest.

## Publisher’s note

All claims expressed in this article are solely those of the authors and do not necessarily represent those of their affiliated organizations, or those of the publisher, the editors and the reviewers. Any product that may be evaluated in this article, or claim that may be made by its manufacturer, is not guaranteed or endorsed by the publisher.
