# Supplementary material for: Trends in metabolic signaling pathways of tumor drug resistance: A scientometric analysis
Source: Front Oncol. 2022 Oct 25;12:981406. doi: 10.3389/fonc.2022.981406 (PMC9641273; doi:10.3389/fonc.2022.981406)
Supplement: Supplementary file 1 [file DataSheet_1.pdf]

Supplementary material

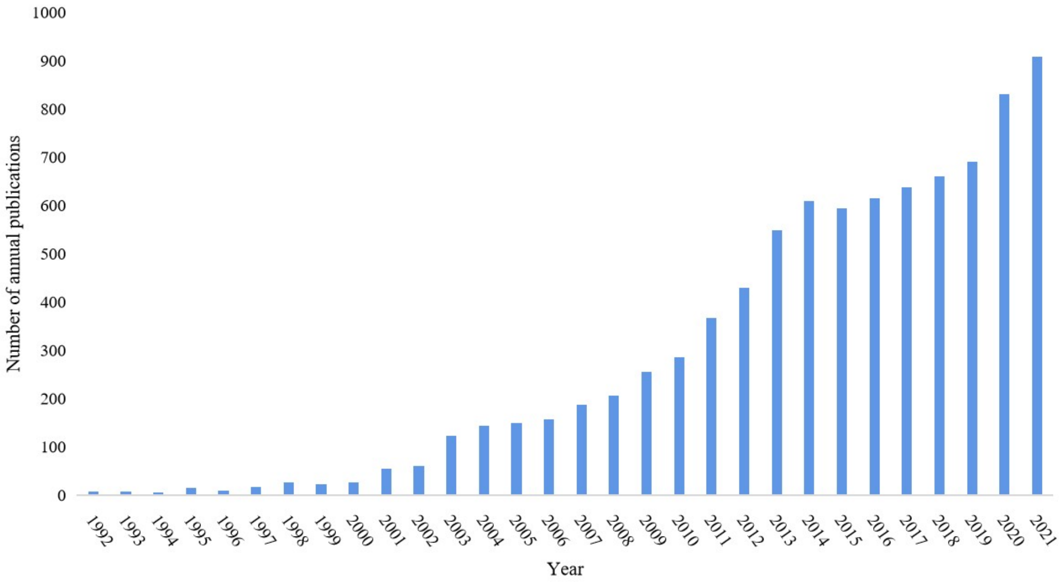

Figure S1 The changing trend of annual publications of published papers of PubMed

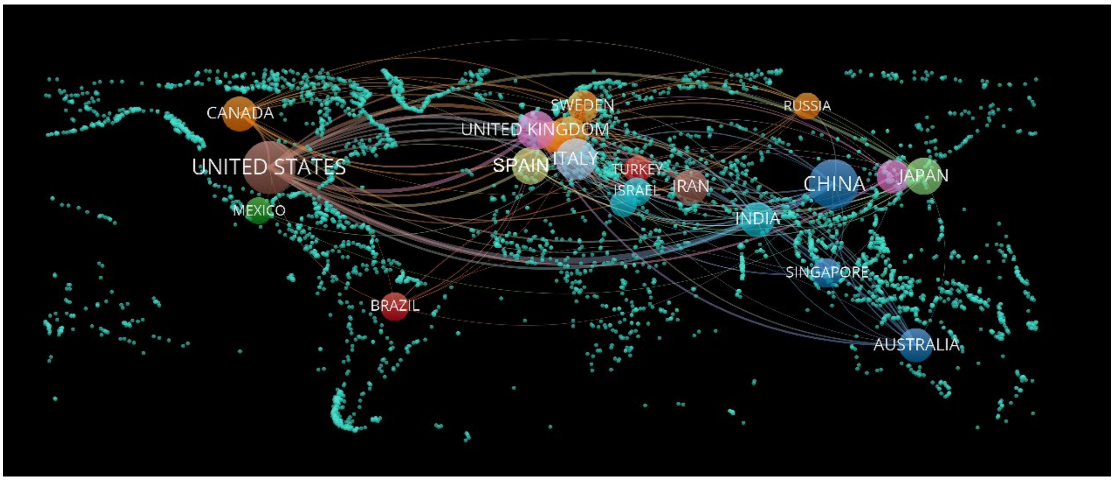

Figure S2 Geographic country/regions co-authorship map

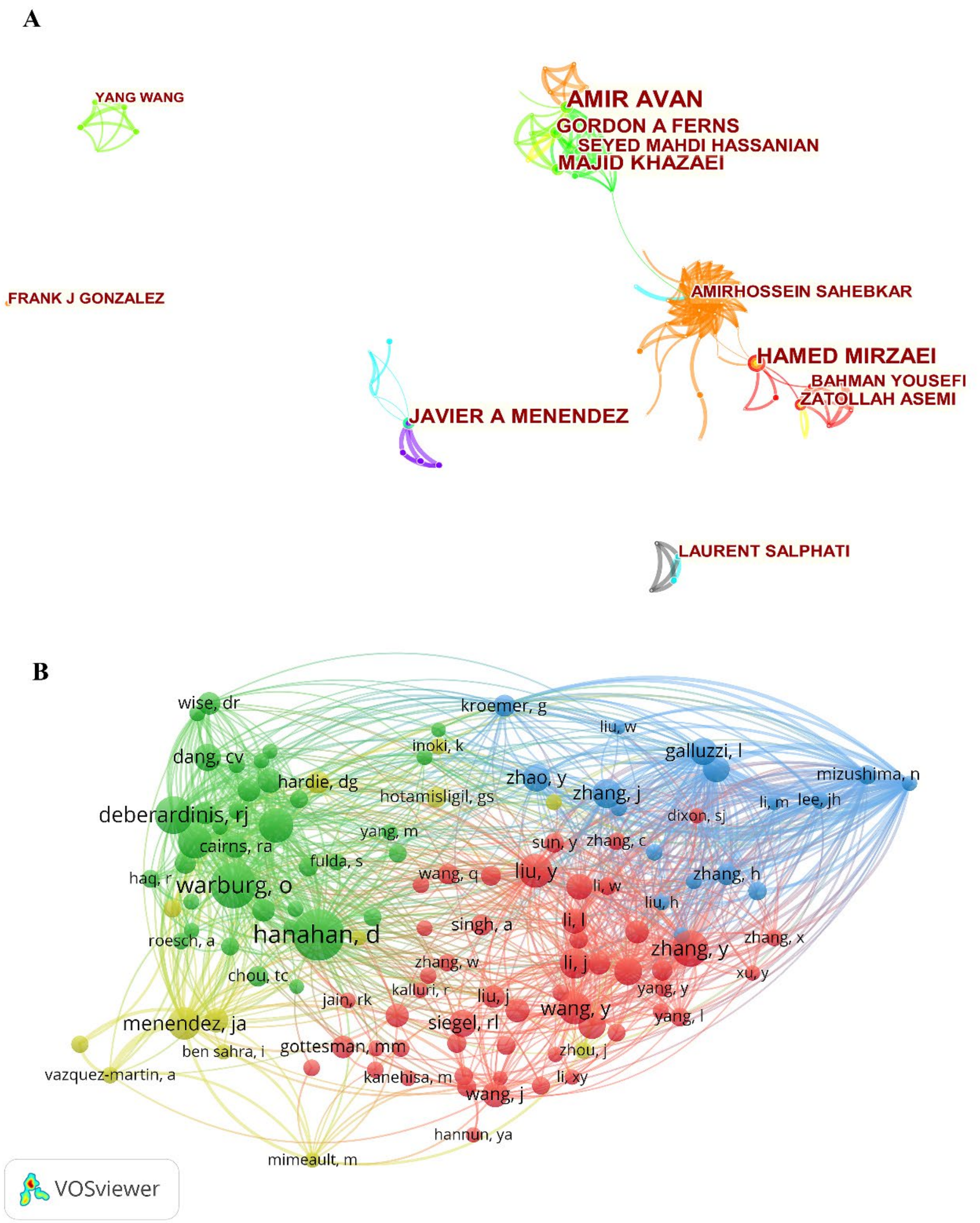

Figure S3| (**A**) Visualization map of co-authorship analysis based on Citespace. (**B**) The co-citation analysis of the authors carried out by using VOS viewer. The color of the nodes and lines indicated different appearance clusters. The node size is proportional to citation frequency. A line between two nodes indicates that both were cited by one author.

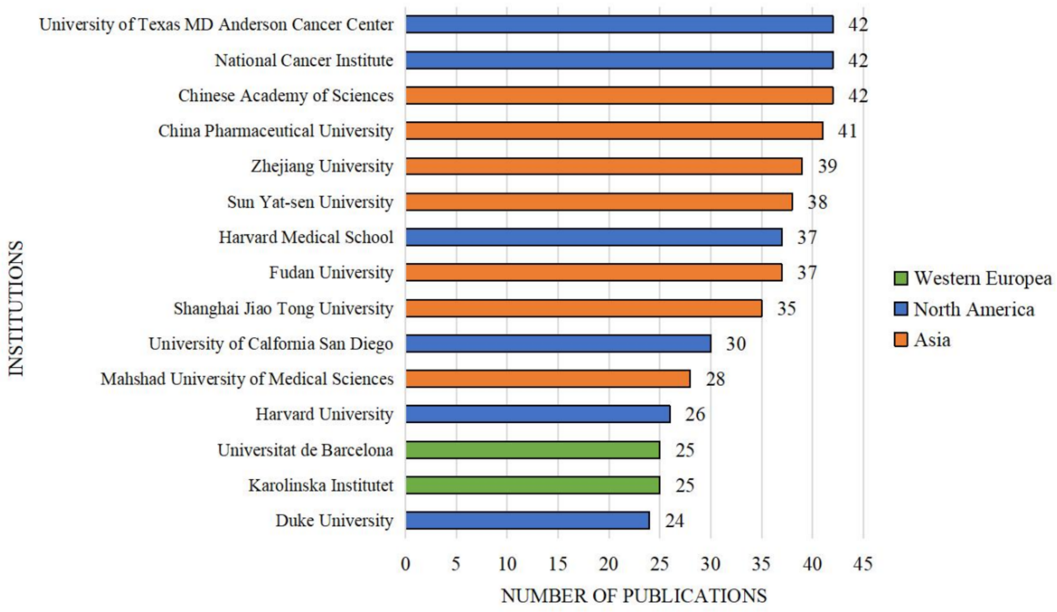

Figure S4 The top 15 institutions with the most publications of metabolic signaling pathways of tumor drug resistance research.

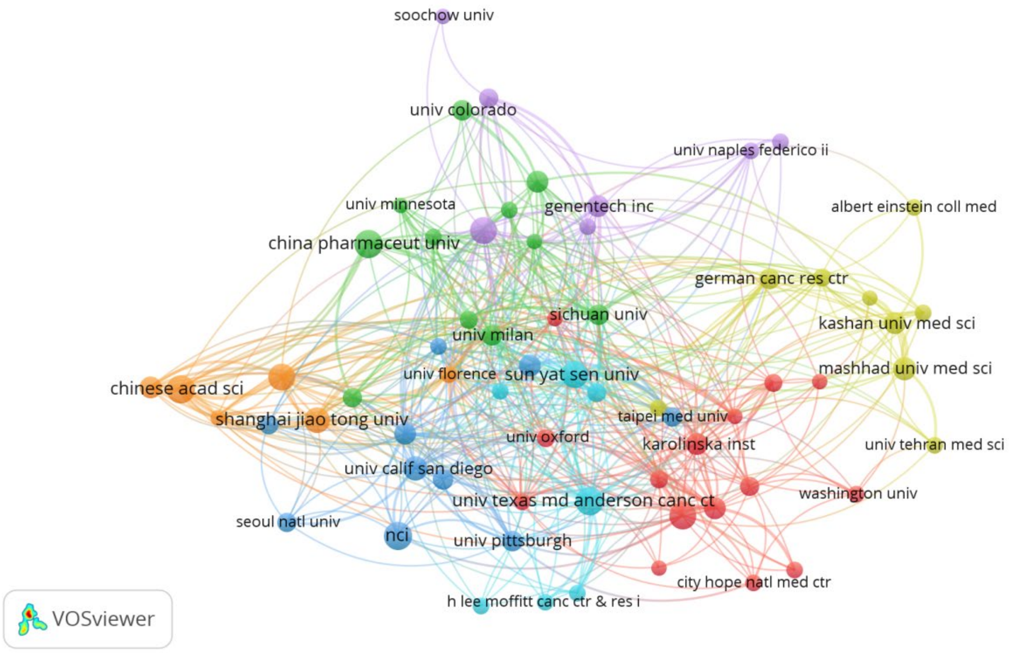

Figure S5 Institution co-authorship analysis was performed with VOS viewer.

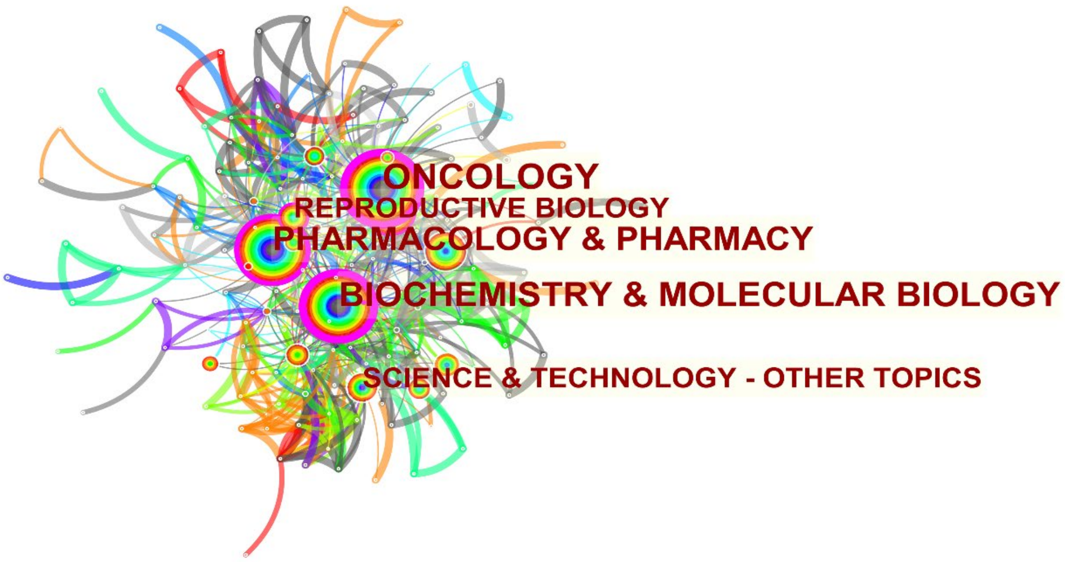

Figure S6 Co-occurring subject categories network using CiteSpace.

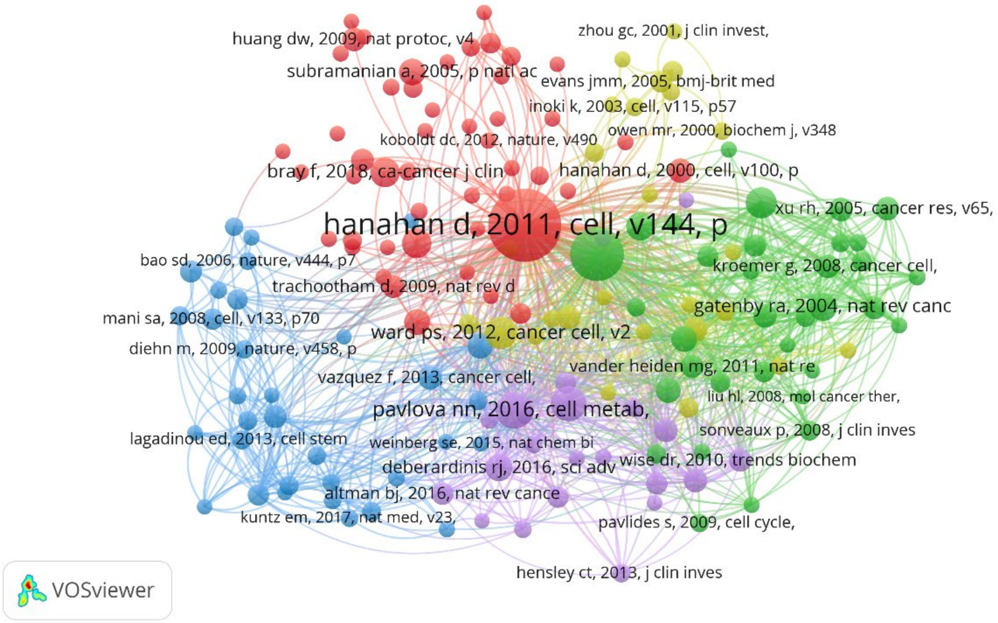

Figure S7 Co-citation network visualization map of references using VOS viewer. The color of the nodes and lines indicated different appearance clusters. The node size is proportional to citation frequency. A line between two nodes indicates that both were cited by one reference.

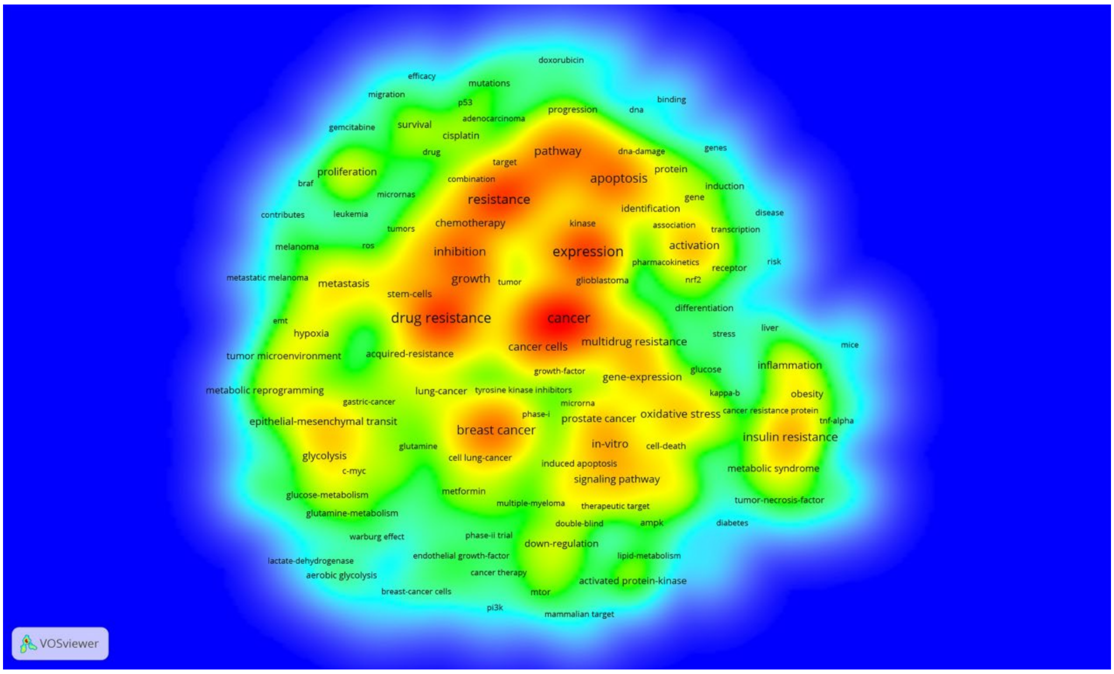

Figure S8 A density visualization map of included keywords using VOS viewer. The depth of the color was positively correlated with the occurrences of keywords.

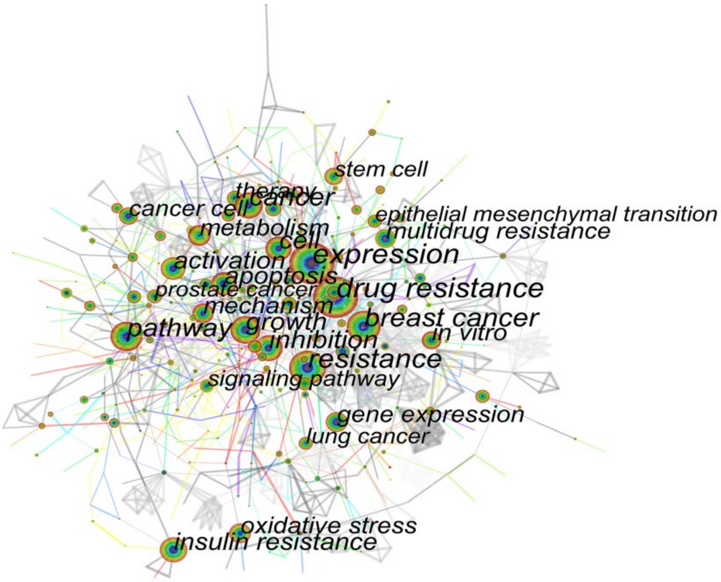

Figure S9 A visualization knowledge map of high-frequency keywords generated by Citespace.

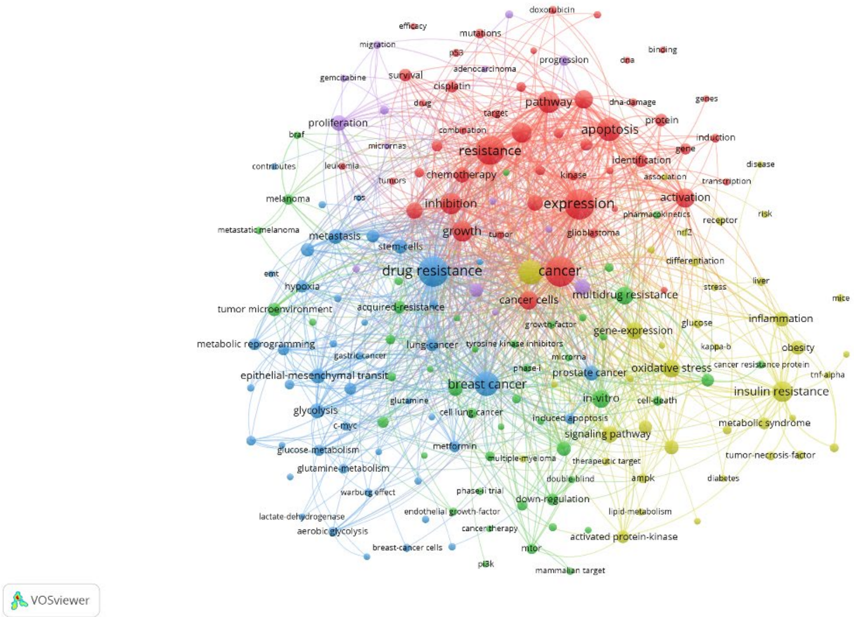

Figure S10 Network visualization map of keyword co-occurrence analysis using VOS viewer. All keywords are labeled. The size of the node reflects the occurrence frequency of a certain keyword. The larger the size of the node is, the more frequently the keyword co-occurs. VOS viewer marks keywords with different colors, and the color of the nodes and labels indicates the cluster in which they belong to. Closely related keywords are grouped into one cluster with the same color. The higher the quantity of co-occurrences of two keywords, the closer will they be located in the network.

Table S1 The top 10 productive journals that published articles on metabolic signaling pathways of tumor drug resistance

Ranking	Journal	Country	Output [n (%)]	IF (2021)	Quartile in category (2021)
1	Cancers	Switzerland	106 (4.18%)	6.575	Q1
2	Frontiers in Oncology	Switzerland	74 (2.92%)	5.738	Q2
3	International Journal of Molecular Sciences	Switzerland	61 (2.40%)	6.208	Q1
4	Plos One	USA	51 (2.01%)	3.752	Q2
5	Oncotarget	USA	48 (1.89%)	N/A	N/A
6	Cancer Research	USA	33 (1.30%)	13.312	Q1
7	Scientific Reports	England	32 (1.26%)	4.996	Q2
8	Frontiers in Pharmacology	Switzerland	28 (1.10%)	5.988	Q1
9	Cell Death & Disease	England	27 (1.06%)	9.705	Q1
10	Molecular Cancer Therapeutics	USA	26 (1.02%)	6.009	Q2

IF: Impact factor

Table S2 The top 20 productive authors from 1992 to 2022

Ranking	Author	Output	Country/Region
1	Avan A	19	Iran
2	Mirzaei H	14	Iran
3	Gonzalez FJ	13	USA
4	Khazaei M	13	Iran
5	Ferns GA	11	UK
6	Wang GJ	11	China
7	Hassanian SM	10	Iran
8	Huang P	10	China
9	Asemi Zatollah	8	Iran
10	Riganti C	8	Italy
11	Salphati L	8	USA
12	Zhang W	8	China
13	Zhou F	8	China
14	Krausz KW	7	USA
15	Pang J	7	USA
16	Shahidsales Soodabeh	7	Iran
17	Giovannetti Elisa	6	Netherlands
18	Hamblin MR	6	USA
19	Huang Min	6	China
20	Li Li	6	China

Table S3 The top 20 co-cited authors from 1992 to 2022

Ranking	Co-cited author	Citations	TLS
1	Hanahan D	353	2,688
2	Warburg O	290	2,395
3	Deberardinis RJ	215	2,220
4	Zhang Y	202	2,294
5	Semenza GL	187	1,821
6	Heiden MGV	182	1,621
7	Liu Y	181	1,795
8	Wang Y	171	1,566
9	Menendez JA	170	1,844
10	Li J	154	1,273
11	Zhang J	143	1,699
12	Wang L	133	1,375
13	Dang CV	131	1,406
14	Li Y	131	1,043
15	Galluzzi L	128	2,191
16	Siegel RL	127	489
17	Zhao Y	127	1,381
18	Kim J	124	2,325
19	Wang J	119	1,107
20	Zhang L	119	1,110

TLS: Total link strength

Table S4 The top 20 authors with the strongest citation bursts from 1992 to 2022

Author	Strength	Begin	End	1992 - 2022
Jemal A	13.7	2007	2016	
Gatenby RA	11.61	2006	2017	
Laplante M	11.54	2016	2018	
Engelman JA	10.9	2008	2014	
Heiden MGV	10.59	2011	2017	
Gottesman MM	10.05	2003	2016	
Siegel R	9.71	2014	2017	
Pelicano H	9.3	2014	2017	
Zhao Y	9.04	2014	2017	
Dang Cv	9.04	2012	2018	
Kroemer G	9	2009	2017	
Bray F	8.9	2020	2022	
Shaw RJ	8.85	2010	2018	
Fantin VR	8.74	2007	2018	
Ward PS	8.69	2014	2017	
Flaherty KT	8.31	2012	2017	
Xu RH	7.89	2007	2015	
Kaelin WG	7.84	2013	2017	
Hsu PP	7.79	2016	2018	
Chapman PB	7.5	2015	2019	

Table S5 The top 25 references with the strongest citation bursts from 1992 to 2022

References	Strength	Begin	End	1992 - 2022
Pavlova NN(55)	38.33	2017	2022	
Hanahan D(53)	34.11	2011	2016	
Bray F(58)	33.67	2019	2022	
DeBerardinis RJ(97)	25.42	2018	2022	
Liberti MV(98)	23.98	2019	2022	
Ward PS(99)	18.41	2014	2017	
Zhao Y(100)	18.34	2014	2018	
Altman BJ(101)	18	2017	2022	
Vander Heiden MG(102)	17.35	2019	2022	
Siegel RL(59)	16.22	2019	2022	
Saxton RA(103)	14.99	2018	2022	
Heiden MGV(50)	14.37	2011	2014	
Lee KM(56)	14.36	2019	2022	
Cairns RA(104)	13.29	2012	2016	
Kuntz EM(105)	13.26	2018	2022	
Sancho P(106)	13.23	2017	2020	
Holohan C(66)	13.1	2016	2018	
Haq R(44)	12.81	2015	2018	
Siegel RL(60)	12.67	2015	2019	
Pascual G(107)	12.3	2019	2022	
Siegel RL(108)	12.13	2018	2020	
Viale A(109)	11.51	2015	2019	
Zhang G(110)	10.92	2017	2020	
Wang TY(57)	10.75	2020	2022	
Laplante M(111)	10.74	2015	2017	
